# The essential role of tumor suppressor gene *ING4* in various human cancers and non-neoplastic disorders

**DOI:** 10.1042/BSR20180773

**Published:** 2019-01-30

**Authors:** Yang Du, Yan Cheng, Guanfang Su

**Affiliations:** Department of Ophthalmology, The Second Hospital of Jilin University, 218 Ziqiang Street, Changchun, Jilin 130041, China

**Keywords:** aberrant expression status, chromosome remodeling, gene therapy, inhibitor of growth 4, mechanisms, non-neoplastic diseases

## Abstract

Inhibitor of growth 4 (ING4), a member of the ING family discovered in 2003, has been shown to act as a tumor suppressor and is frequently down-regulated in various human cancers. Numerous published *in vivo* and *in vitro* studies have shown that ING4 is responsible for important cancer hallmarks such as pathologic cell cycle arrest, apoptosis, autophagy, contact inhibition, and hypoxic adaptation, and also affects tumor angiogenesis, invasion, and metastasis. These characteristics are typically associated with regulation through chromatin acetylation by binding histone H3 trimethylated at lysine 4 (H3K4me3) and through transcriptional activity of transcription factor P53 and NF-κB. In addition, emerging evidence has indicated that abnormalities in ING4 expression and function play key roles in non-neoplastic disorders. Here, we provide an overview of ING4-modulated chromosome remodeling and transcriptional function, as well as the functional consequences of different genetic variants. We also present the current understanding concerning the role of ING4 in the development of neoplastic and non-neoplastic diseases. These studies offer inspiration for pursuing novel therapeutics for various cancers.

## Introduction

Tumor suppressor genes (TSGs) can oppose oncogene function and restrain cancer development, with inactivation of TSGs being essential for cancer development, along with the aberrant activation of oncogenes [1]. The inhibitor of growth (ING) family consists of five homologous proteins [2], ING1–ING5, which function as the type II tumor suppressor. Shiseki et al. [3] state that they first cloned the ING4 cDNA from human placenta cDNA library in 2003. The *ING4* gene is located at chromosome 12p13.31, and includes eight exons spanning over a 13-kb genomic interval [4]. ING4 cDNA generated from the processed ING4 mRNA consists of 1380 nts encoding a 29-kDa nuclear protein comprising 248 or 249 amino acids [3,4]. ING4 is ubiquitous in multiple human tissues and is frequently mutated in various cancer cell lines [5,6]. Decreased expression or dysregulation of ING4 is widely seen in diverse types of cancers. However, ING4-null mice fail to show increased spontaneous tumor formation, suggesting that ING4 deficiency by itself may not be sufficient to initiate tumorigenesis [7]. This review summarizes the current literature on ING4, and efforts in the advancement of potential clinical gene therapy strategies for cancers.

## Structure of the ING4 protein

Several regions within the ING4 protein are indispensable for its proper function. The N-terminal region of ING4 is folded into a coiled-coil domain, forming an antiparallel dimer [8,9]. Homology in ING family is the highest at the carboxyl termini within a plant homeodomain (PHD) finger. The PHD motif is common to many chromatin regulatory proteins with binding sites for the histone H3 trimethylated at lysine 4 (H3K4me3) peptide [10,11]. The two PHD fingers contain C4HC3-type zinc fingers spanning 50–80 amino acid residues [12], and point in opposite directions when the protein is stretched [13].

The central region ING4, containing approximately 85 residues, behaves as a disordered random coil. As this region is rich in basic amino acids and has a potential bipartite nuclear localization signal (NLS) domain, it is commonly referred to as the NLS region. It is essential for nuclear localization and a second NLS cluster (RARSK) is required for the binding of ING4 to p53 [14]. Peptidyl arginine deiminase 4 (PAD4) preferentially citrullinates ING4 in the RARSK region, thereby disrupting the interaction between ING4 and p53 [15]. This is likely because of the highly disordered nature of the NLS region of ING4 [16]. Mutational studies have identified two intrinsic nucleolar translocation sequences (NTSs) within the NLS region of ING4 [17]. Mediated by NTS, ING1 translocates to the nucleolus after UV-induced DNA damage [17]. Three potential NTS motifs—RRQR, KEKK, and KKKK—are well conserved only in ING1 and ING2, but are missing from ING4, suggesting that the ability of ING4 to be targetted to the nucleolus in response to UV-induced DNA damage is compromised or lacking [18].

Amino acid sequence alignment of ING4 proteins reveals several other conserved regions, including a leucine zipper-like (LZL) motif and novel conserved region (NCR). The LZL motif consists of leucine residues at every seventh amino acid at the N-terminus of ING2, forming a hydrophobic patch [18]; it is responsible for DNA repair, apoptosis, and chromatin remodeling after UV irradiation [19]. A similar leucine distribution is present in ING4, which suggests potentially similar functions [18], in addition to mediating protein dimerization [8]. The NCR of ING4 is also known as the lamin interaction domain (LID), owing to its capacity to interact with endogenous lamin A [20].

## Genetic splice variants

Alternative mRNA splicing and the coding sequences of alternative splice variants play an important role in the expansion of proteome diversity by the production of multiple protein isoforms [21]. Eight splice variants of ING4 have been reported thus far [22–25]. Different variants have different functions and the balance of expression of these variants may confer greater diversity to the functions of ING4 in tumorigenesis, since some of the variants can have dominant negative effects. Moreover, these variants are also detected in normal tissue samples; therefore, it may be reasonable to assume that they contribute to normal development in addition to neoplasia. The original ING4 (ING4_v1), the longest ING4 splice variant, encodes an intact NLS and negatively affects cell proliferation, contact inhibition, and angiogenesis. This variant can interact with several cytoplasmic proteins, including Liprin α1 and G3BP2 [23]. Unoki et al. [23] identified three additional ING4 variants (ING4_v2, ING4_v3, and ING4_v4) by an expression sequence tag (EST) search using the Basic Local Alignment Search Tool (BLAST) program. ING4 contains GC(N)7GT and NAGNAG motifs at the exon 4–5 boundary, which could cause canonical (GT-AG) and non-canonical (GC-GT) splicing-site wobble selection [25,26]. ING4_v2 (a 3-bp skip type) as well as the splice variants ING4_v3 and ING4_v4 lack a full NLS, resulting in increased cytoplasmic localization of these proteins. Therefore, these proteins may function incorrectly. As a result, ING4_v2 retains a suppressive effect on cell migration; however, it has lost its suppressive effect on cell spreading. In contrast, despite disruption of the NLS domain by alternative splicing, Tsai et al. [25] found that the four splicing isoforms maintain their nuclear localization. ING4_v3 (a 9-bp skip type) is expressed at relatively low levels and is degraded rapidly. Therefore, it may be the product of aberrant splicing *in vivo* [25]. ING4_v4 (a 12-bp skip type) is reportedly attributed to a common deletion mutation [5]; it exerts a dominant negative effect in the induction of p21^WAF1^ promoter activation and in the suppression of cell spreading and migration by ING4-v1. These ING4 variants are expressed ubiquitously in various tissues, but are maintained at low levels, except for in the brain and testis. In addition, serum starvation/activation or DNA-damaging reagents, such as adriamycin and etoposide, fail to differentially induce type-specific expression of the variants. Four other novel ING4 variants that lack exons 2, 3, and 6 are named ING4-ΔEx2, ING4-ΔEx3, ING4-ΔEx6A, and ING4-ΔEx6B, respectively [24]. All ING4 variants retain the ability for nuclear localization, interaction with p53, and formation of the histone acetyltransferase (HAT)/histone acetyltransferase binding to ORC1 (HBO1) complex [24]. ING4-ΔEx6A does not express PHD and has lost the ability to inhibit nuclear factor κB (NF-κB) activation [24,27].

## Chromosome remodeling and transcriptional function

Regulation of gene expression is inherently associated with alterations in chromatin architecture. Patterns of covalent modifications of DNA and histones, described as epigenetic factors, influence chromatin structure, genome stability, and gene expression, all of which are fundamental to the cellular changes that are responsible for the development of cancer [28]. Of significance are post-translational modifications (PTMs) achieved by phosphorylation, methylation, and deacetylation of nucleosomal histones within the promoters, which give rise to the silencing of TSGs in cancers [29–32]. Amongst the histone modification repertoire, histone acetylation, governed by HATs and histone deacetylases (HDACs), is crucial for transcriptional regulation and DNA repair, recombination, and replication. ING4 has been shown to co-purify with the HBO1/JADE/hEaf6 HAT complex, which is responsible for the majority of histone H4 acetylation. Depletion of ING4 from the complexes specifically affects their ability to acetylate nucleosomal histones and to efficiently modify chromatin [33–35]. With respect to the complexes, the ING4 PHD finger specifically recognizes H3K4me3, which augments HBO1 acetylation activity on H3 tails and drives H3 acetylation of promoters targetted by ING4 [8,9,35,36]. Extensive evaluation of the crystal structure of the ING4 N-terminal domain has revealed that this domain forms an antiparallel coiled-coil homodimer, with each protomer folding into a helix–loop–helix structure, and the two PHDs of the ING4 dimer independently binding to the H3K4me3 peptides with equal affinity [8,9]. Recently, it was recognized that the positively charged NLS region enables the preferential binding of ING4 with dsDNA through micromolar affinity, which favors the recognition and recruitment of the HAT/HBO1 complex to chromatin sites enriched with H3K4me3 [13]. The H3K4me3 recognition by ING4 is compatible with certain histone proteins [11]. However, the interaction of PHD fingers and other protein modules remain unknown; more combinations of modified histones may also exist in this process. Further research in this field will better illuminate the regulatory mechanism of chromatin. Moreover, HBO1-JADE-ING4 complexes, which are enriched near the transcription start site (TSS) of p21/CDKN1A, directly stimulate p53-dependent transcription of genes such as *p21* through the ING4-dependent association with H3K4me near the TSS [37].

*TP53*, encoding the tumor suppressor protein p53, is the most frequently mutated gene in approximately half of the cancers. The p53 protein acts as a sentinel for stress factors and regulates a series of crucial cellular processes [38,39], including cell cycle arrest [3,40,41] and apoptosis [3]. ING4 has been shown to interact with p53 in various cancer types. Shiseki et al. [3] provided the first evidence that ING4 induces the expression of *p21/WAF1*, a well-characterized p53-regulated gene whose promoter contains consensus sequences of the p53-binding sites [42], by activating its promoter. Mechanistically, ING4 physically interacts with the HAT complexes, together with P53 and acetyltransferase E1A binding protein P300, in which exogenous ING4 enhances p53 acetylation at Lys^382^ residues and alters its transcriptional activation. In contrast, Gunduz et al. [4] did not detect such a direct relationship when comparing the p53 mutation status with ING4 expression in head and neck squamous cell carcinomas. To our knowledge, there are two regulators known to influence the interaction between ING4 and P53. The first is EBNA3C, a cancer-promoting protein from Epstein–Barr virus, which binds to the NLS region of ING4 at residues 129–200 at the amino-terminal site, and then competitively blocks p53 binding and subsequent activation [43]. The second is human papillomaviruses 16 early oncoprotein E6 (HPV16 E6), which combines with ING4 to impede p53 acetylation by hindering the interaction of p53 and ING4 [44]. It is reasonable to speculate that ING4 may also be a possible target of many other oncogenic viruses. Restoration of ING4 expression could probably show therapeutic significance.

Transcription factor NF-κB is involved in a wide range of functions, in both homeostasis and pathology, especially in human cancers. The canonical pathway of NF-κB activation involves phosphorylation and ubiquitin-proteasome-mediated degradation of inhibitor of κB (IκB), resulting in the release of NF-κB subunits from the cytoplasmic IκB complex [45]. Subsequently, p65 (RelA)/p50 heterodimers translocate into the nucleus and bind to gene promoter sequences and induce target gene expression [46,47]. ING4 has been reported to trigger NF-κB effects in multiple cancers, through the abovementioned mechanism [27,48–54]. As first shown by Garkavtsev et al. [55], ING4 interacts with the p65/RelA subunit of NF-κB and subsequently regulates the growth of transplanted glioblastoma through transcriptional repression of NF-κB-responsive genes. Overexpression of ING4 has been shown to inhibit phosphorylation of p65/RelA at the amino acid residue Ser^536^. Phosphoactivation of p65/RelA, along with the levels of acetylated histones and H3K4me3, helps to explain how ING4 suppresses NF-κB-regulated promoters [56,57]. Moreover, together with the E2 enzyme UbcH3, ING4 is found to promote K48-linked ubiquitination and proteasomal destruction of p65 via its PHD motif, which targets the Lys^62^ residue of p65 [58]. On the other hand, ING4 positively regulates IκB promoter activation, thereby suppressing nuclear RelA levels and the activation of NF-κB signaling [7].

Expression of the MYC proto-oncogene is deregulated in a variety of cancer types. The Myc oncoproteins belong to a family of so-called ‘super-transcription factors’ that potentially regulate a broad range of biological functions, such as cell proliferation, cell differentiation, cell survival, and immune surveillance [59,60]. Expression of a dominant negative mutant of ING4 has been considered to co-operate with the MYC oncogene to form mammary tumors [61]. AU-rich RNA-binding factor 1 (AUF1) is a protein reported to promote MYC translation through binding to the MYC ARE motif [62]. ING4 can impair pro-oncogene c-myc translation via interaction with AUF1 and abolish cell proliferation in human chronic myeloid leukemia cells [63]. Berger et al. [64] propose a mechanism in which loss of ING4 promotes Myc-driven oncogenesis of prostate cells by deregulating epithelial differentiation. Nevertheless, Myc has been shown to bind to the ING4 promoter, suggesting that ING4 is a direct target of Myc in medulloblastoma [65].

## Dysregulation of ING4 in tumors and mechanisms of tumor suppression

**Figure 1:**
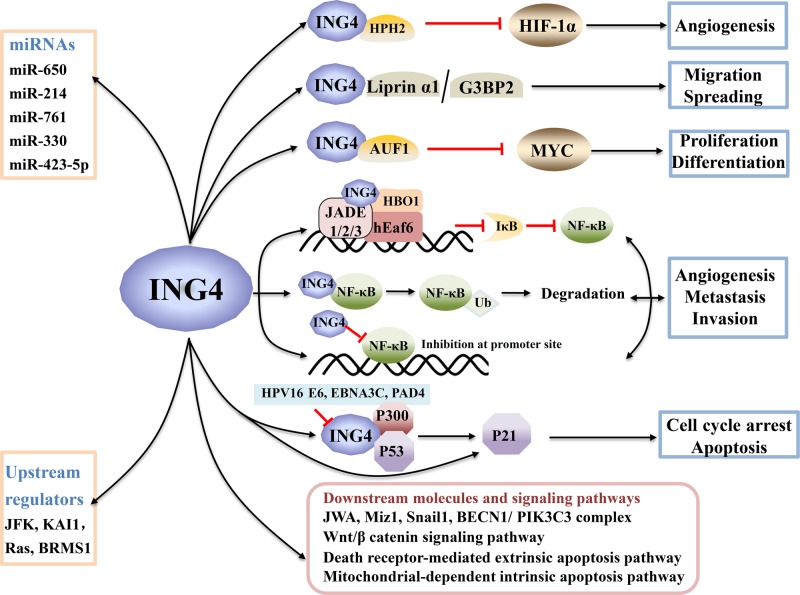
The known mechanisms of ING4 transcriptional regulation and its suppression of tumor growth and progression through downstream targets to modulate cellular events

Suppressive properties of ING4 were widely studied and confirmed by various research groups. Compelling evidence suggests that the human ING4 protein is highly expressed in normal tissues, but that its expression is dramatically decreased in some types of cancer (Table 1), suggesting that its aberrant expression may contribute to the pathogenesis of these cancers. Based on changes in expression, a six-biomarker system (composed of ING4 and five additional biomarkers) provides a more accurate prognosis for melanoma patients than any single biomarker [66]. ING4 expression is also considered to be a significant biomarker that may be used to discriminate melanoma from dysplastic nevi [67]. ING4 can drive prostate luminal epithelial cell differentiation by targetting Miz1 [68], since dysregulated differentiation is implicated in prostate oncogenesis [64]. For a better understanding of the role of ING4 in pathogenesis, we focussed on the mechanisms underlying ING4-related tumor suppression. The known mechanisms of ING4-mediated suppression of tumorigenesis are depicted in Figure 1.

**Table 1:** Aberrant expression status of ING4 and functional impacts in human cancers reported by different research groups

Cancer types	Origin	Methods of detection	Alteration	Frequency	Putative or observed functional consequences	Transcripts dysregulation	Refs
Ameloblastoma	Patients	MM	Loss of heterozygosity (LOH)	11/29 (37.9%) (ING4MS1)			[69]
				10/30 (33.3%) (ING4MS2)			
Astrocytomas	Patients	IHC	Up-regulation	7/101 (6.9%)	Tumor grade	NF-κB (p65), MMP-2, MMP-9, u-PA	[49]
	Patients	IHC	No change	72/101 (72.3%)			
	Patients	IHC	Down-regulation	21/101 (20.8%)			
Bladder cancer	Patients	WB, IHC, RT-qPCR	Down-regulation	Not mentioned			[70]
Breast cancer	Patients	IHC	Down-regulation	77/227 (34%)	Tumor grade, Lymph node status	NF-κB (p65)	[57]
	Cell lines	WB	Down-regulation	1/1	Cell invasion	NF-κB (p65), IL6, IL8, and PTGS2 (COX2)	
	Patients	RT-PCR, WB, IHC	Down- regulation	38/60 (53.3%)	Microvessel density		[71]
	Patients	FISH	Gene locus deletion	170/1033 (16.5%)	Suppressive role in the HER2-driven oncogenesis		[72]
	Patients and cell lines	Comparative genomics	Gene locus deletion	10–20%	Suppresses loss of contact inhibition and growth	Induced by MYC-family oncogenes	[5]
		Hybridization (CGH)					
	Cell lines	Q-PCR	Down-regulation	3/9 (ING4v1)			[73]
				2/9 (ING4v2)			
	Patients	IHC	Cytoplasmic expression > nucleus expression	67/70 (95.7%)	More aggressive and metastatic potential	HER2+	[74]
Breast carcinomas	Patients	WB	Down-regulation	11/15 (73.3%)	Negatively correlated with JFK		[53]
Cervical cancer	Patients	WB, IHC, RT-qPCR	Down-regulation	18/18 (100%)			[75]
Chronic lymphocytic leukemia	Patients	RNAseq, RT-qPCR	Up-regulation	6/6 (100)	Cell cycle regulation, checkpoint, and centromere function		[76]
Clear cell renal carcinoma	Patients	WB, RT-qPCR	Down-regulation	40/40 (100%)	Nuclear grade, clinical stage, lymphatic metastasis		[77]
	Patients	IHC	Cell membrane and cytoplasm	51/62 (82.3%)			
Colon adenocarcinoma	Cell lines	Q-PCR	Down-regulation	0/2 (ING4v1)			[73]
				0/2 (ING4v2)			
Colorectal cancer	Patients	WB, IHC, RT-qPCR	Down-regulation	9/10 (90%)	Lymph node metastasis, advanced TNM stage, poor overall survival		[78]
	Cell lines	RT-qPCR	Down-regulation	4/4	Tumor growth, invasion and metastasis, microvessel density	P21, E-cadherin, cyclin E, IL-6, IL-8, VEGF, Snail1, N-cadherin, vimentin	[79]
Colorectal carcinoma	Patients	RT–qPCR, WB, IHC	Down-regulation	22/60 (36.7%)	Microvessel density		[80]
	Patients	IHC	Down-regulation	25/97 (25.8%)	Dukes’ stages, lymphatic metastasis		[81]
Gastric adenocarcinoma	Patients	RT-PCR	Down-regulation	30/40 (75%)	Tumor grade		[82]
	Patients	RT-qPCR	Down-regulation	13/13 (100%)			
	Patients	IHC	Down-regulation	29/40 (72.5%)			
	Patients	WB	Down-regulation	3/5			
	Patients	TM-IHC	Down-regulation	99/120 (82.5%)			
	Cell lines	WB	Down-regulation	3/4			
Gastric carcinoma	Patients	ISH	Down-regulation	62/85 (72.9%)	Proliferation and invasion	NF-κB (p65), p-IκBa, IκBa, MMP-9, uPA	[27]
	Patients	IHC	Down-regulation	59/85 (69.4%)			
	Patients	RT-qPCR	Down-regulation	10/10 (100%)			
	Cell lines	RT-qPCR	Down-regulation	3/3			
	Cell lines	WB	Down-regulation	3/3			
Gastrointestinal stromal tumor	Patients	IHC	Down-regulation	24/41 (58.5%)	Tumor size, mitotic index, tumor necrosis, invasion, recurrence and metastasis, mortality		[83]
Glioblastomas	Patients	WB	Down-regulation	12/24 (50%)		Interact with miR-423-5p	[84]
Glioma	Patients	RT-qPCR, IHC	Down-regulation	50/50 (100%)	Tumor grade		[55]
	Patients	WB, IHC	Down-regulation	11/14 (78.6%)	Tumor grade		[56]
	Patients	IHC	Down-regulation	49/60 (81.7%)	Tumor grade		[85]
	Patients	RT-PCR	Down-regulation	15/15 (100%)			
	Patients	SQRT-PCR, WB	Down-regulation		Pathological grade, microvessel density, cell proliferation index		[86]
	Patients	IHC	Down-regulation	69/85 (81.2%)			
Head and neck squamous cell carcinoma	Patients	IHC	Low nuclear expression	96/214 (44.9%)	Differentiation, T stage, and TNM stage, lymph node metastasis	14-3-3η, p300, P21	[87]
	Patients	IHC	High cytoplasmic expression	143/214 (66.8%)			
	Cell lines	RT-PCR, sequencing	Similar expression, no mutation	3/3			
	Cell lines	WB, IHC	Low nuclear expression	2/3			
	Cell lines	WB, IHC	High cytoplasmic expression	1/3			
Head and neck squamous cell carcinoma	Patients	MM	LOH	33/50 (66%)			[4]
	Patients	Sequencing	No mutation	0/50			
	Patients	RT-qPCR	Down-regulation	38/50 (76%)			
	Patients	RT-qPCR	Up-regulation	7/50 (14%)			
Hepatocellular carcinoma	Patients	RT-qPCR, WB	Down-regulation	19/36 (52.8%)	Edmondson–Steiner grade, vein invasion, microvessel density		[88]
	Patients	IHC	Down-regulation	65/136 (47.8%)			
	Patients	WB	Down-regulation	5/8 (62.5%)		Interact with miR-650	[89]
Lung adenocarcinoma	Patients	RT-PCR	Down-regulation	20/20 (100%)	Differentiation degree		[90]
	Patients	IHC, RT-qPCR, WB	Down-regulation	11/18 (61.1%)	Docetaxel chemoresistance	Bcl-2/Bax, caspase-3	[91]
	Cell lines		Down-regulation	2/2			
	Cell lines	WB	Down-regulation	2/2	Docetaxel chemoresistance	Interact with miR-650	[92]
Lung cancer	Patients	SQRT-PCR, WB	Down-regulation	50/50 (100%)	Lymph node metastasis (nuclear expression), tumor grade (nuclear and cytoplasmic expression)		[93]
	Patients	IHC	Low cytoplasmic expression	201/246 (81.7%)			
	Patients	IHC	Low nuclear expression	217/246 (88.2%)			
	Patients	IHC	Cytoplasmic expression > nucleus expression	110/246 (44.7%)			
	Patients	IHC	Cytoplasmic expression < nucleus expression	28/246 (11.4%)			
Lung carcinoma	Cell lines	Q-PCR	Down-regulation	2/2 (ING4v1) 2/2 (ING4v2)			[73]
Melanoma	Patients	IHC	Down-regulation	10/17			[94]
Melanoma	Patients	IHC	Down-regulation	34% (primary)	Tumor thickness, ulceration, poor survival outcome with primary melanoma		[95]
		IHC	Down-regulation	47% (metastatic)			
Multiple myeloma	Cell lines	RT-qPCR	Down-regulation	8/8	Tumor angiogenesis	HIF-1α, IL-8, OPN	[48]
Non-small cell lung cancer	Patients	WB, IHC	Down-regulation	28/28			[96]
Osteosarcoma	Patients	IHC	Down-regulation	26/41 (63.4%)	Negatively correlated with Enneking classification		[97]
Ovarian cancer	Patients	SQRT-PCR	Down-regulation	23/40 (57.5%)	Clinical stage, histological grade, microvessel density		[71]
		IHC	Down-regulation	35/40 (87.5%)			
Ovarian carcinoma	Cell lines	Q-PCR	Down-regulation	1/1 (ING4v1)			[73]
				1/1 (ING4v2)			
Pancreatic adenocarcinoma	Cell lines	Q-PCR	Down-regulation	1/1 (ING4v1)			[73]
				1/1 (ING4v2)			
Prostate adenocarcinoma	Cell lines	Q-PCR	Down-regulation	1/1 (ING4v1)			[73]
				1/1 (ING4v2)			
Prostate cancer	Patients	IHC	Down-regulation	32/50 (64%)			[64]

Abbreviations: Bcl-2, B-cell lymphoma-2; FISH, fluorescent *in situ* hybridization; IL, interleukin, ISH, *in situ* hybridization; MM, microsatellite marker; MMP, matrix metalloproteinase; OPN, osteopontin; PTGS2 (COX2), prostaglandin-endoperoxide synthase 2 (cyclooxygenase-2); SQRT-PCR, semi-quantitative RT-PCR; TMA-IHC, tissue microarray and immunohistochemistry; u-PA, urokinase plasminogen activator; VEGF, vascular endothelial growth factor.

### Inhibition of angiogenesis, metastasis, and invasion

Tumor cells generally require the expansion of a vascular network to maintain the necessary oxygen and nutrient levels needed for their rapid growth. Angiogenesis, the formation of new blood vessels from a pre-existing vasculature, provides an appropriate microenvironment for tumor growth [98]. Furthermore, angiogenesis is strongly associated with the increased tumor invasiveness and metastatic potential of this complex biological process. Garkavtsev et al. [55] initially revealed that ING4 physically interacts with the p65/RelA subunit of NF-κB, forming a transcriptional complex that represses NF-κB-responsive genes such as interleukin (IL) 6 (*IL-6*), *IL-8, cox-2*, and *CSF-3* in human glioblastoma U87MG cells. In this way, ING4 affects tumor angiogenesis and directly influences brain tumor growth [55]. ING4 is also reportedly involved in the angiogenic switch in the progression of multiple myeloma and affects the production of the proangiogenic molecules IL-8 and osteopontin (OPN) by inhibiting hypoxia inducible factor-1 α (HIF-1α) under hypoxic conditions [48]. The angiogenesis-related genes *IL-6, IL-8, cox-2, u-PA, Ang-1*, and vascular endothelial growth factor (*VEGF*) have been determined to be regulated by ING4 in multiple tumor cells [51,78,79,90,99–101]. The ING4 protein level has been shown to correlate with vein invasion and microvessel density in several solid tumor tissues [71,80,86,88]. Moreover, in tumor-bearing athymic mice, intratumoral injections of adenovirus-mediated *ING4* (Ad-ING4) suppressed tumor growth and reduced tumor microvessel formation [79,99,100,102]. In a melanoma model, the ability to form tubular structures was consistently strongly inhibited both *in vitro* and *in vivo*. In one trial, BRMS1, a metastasis suppressor, inhibited melanoma angiogenesis by suppressing NF-kB activity and IL-6 expression via induction of ING4 [50]. ING4 can regulate endothelial cell growth and tube formation by activating the promoter of the gene encoding JWA, a protein previously reported to inhibit melanoma cell metastasis. This indicates that the ING4/JWA/ILK signaling pathway may be a promising target for anti-angiogenic therapies [103]. Yan et al. [53] demonstrated that JFK-mediated destabilization of ING4 leads to hyperactivation of the canonical NF-κB pathway and promotes angiogenesis and metastasis of breast cancer; in this process, JFK targets ING4 for ubiquitination and degradation through assembly of a Skp1–Cul1–F-box (SCF) complex.

In tissues of gastrointestinal stromal tumors, the low expression level of ING4 has been found to correlate with tumor risks, in addition to metastasis and invasion [83]. Overexpression of ING4 suppresses cell migration and invasion and significantly reduces the activity of matrix metalloproteinase (MMP)-2 and MMP-9 in melanoma, lung carcinoma, osteosarcoma, and astrocytoma [49,51,95,99]. In other words, ING4 is capable of attenuating NF-κB-mediated cell invasion [27,57]. Liprin α1 is a cytoplasmic protein encoded by *PPFIA1* and is necessary for focal adhesion and axon guidance. ING4 can interact with Liprin α1 to regulate cell migration and prevent invasion and metastasis [104]. Kim et al. described that a COOH-terminal truncation mutant of *ING4* (*ING4*mt14) exacerbates MYC-initiated mammary tumorigenesis by increasing tumor penetrance and metastasis [61]. It was also suggested that the ING4 mutant may be a dominant negative allele that interferes with the tumor-suppression function of the wild-type allele and contributes to tumorigenesis [61]. Additionally, ING4 status correlates with lymph node metastasis in lung, colorectal, breast, and clear cell renal carcinomas [71,77,81,93,105]. According to Tang et al. [106], KAI1 is a potential upstream regulator of ING4 and regulates ING4 at both the transcriptional and protein levels, resulting in the regulation of melanoma cell migration. Subsequently, it was experimentally shown that the regulation of melanoma angiogenesis by KAI1 occurred through the inhibition of blood vessel formation in matrigel plugs, along with the down-regulation of IL-6 and VEGF [107]. The underlying mechanism reportedly involves KAI1 down-regulating the activity of Akt through phosphorylation, which releases the inhibition on ING4 from p65. Epithelial–mesenchymal transition (EMT), a developmental program usurped by cancer cells, enables cells to invade and metastasize [108]. ING4 suppresses tumor invasion and metastasis via reversal of EMT through down-regulation of Snail1 and through a switch from N-cadherin to E-cadherin [79], principally by targetting the Wnt/β catenin signaling pathway [109]. These findings indicate that ING4 may promote metastasis by regulating metastasis-associated genes, such as those encoding MMPs. At the same time, it is still necessary to consider the existence of tumor-specific regulatory mechanisms.

### Restoration of intercellular contact inhibition

Contact inhibition is a pivotal process that is deregulated in transformed cells during carcinogenesis. An increase in adhesiveness between malignant and normal cells or a decrease in adhesiveness between malignant cells and the culture surface gives rise to a loss of sensitivity to contact inhibition by malignant cells [110]. The MYC oncogene encodes a transcription factor that has broad-reaching effects on many cellular functions, most importantly in driving cell growth by regulating genes involved in the cell cycle. ING4 does not directly inhibit cellular proliferation, but instead specifically suppresses the loss of contact inhibition elicited by the overexpression of MYC or MYCN [5]. Experiments demonstrate that human breast cancer cells T47D, which are deficient in ING4, display contact inhibition [5]. Meanwhile, *ING4* is not amongst those genes activated by contact inhibition [111], suggesting that functional *ING4* may not be required for contact inhibition to be exhibited. Thus, ING4 may block this tumorigenic event by maintaining the state of contact inhibition through controlling the transcription of some downstream genes, rather than playing a role at the onset of contact inhibition [112]. In addition, with the apparent deficiency of p53 in T47D cells, the effect of ectopic ING4 expression on colony formation in soft agar appears to be independent of P53 function [5].

### Suppression of adaptation to hypoxia

Most solid tumors are hypoxic in nature due to the rapid tumor growth and limited supply of oxygen to internal tissues. Hypoxia in the microenvironment leads to the stabilization of HIF, which acts as a major regulator of metabolic adaptation that significantly contributes to cancer pathogenesis. Consequently, HIF represents an attractive therapeutic target in cancers. HIF is composed of a constitutively expressed subunit HIF-β that is insensitive to O_2_ levels and an oxygen-regulated subunit HIF-α which is mostly degraded under normoxic and physiological conditions [113–115]. Under normoxic conditions, HIF prolyl hydroxylase-2 (HPH-2), an enzyme that belongs to the HPH family, hydroxylates the conserved proline residues within the oxygen degradable domain of HIF-1α [116,117]. Subsequently, this causes the degradation of HIF, leading to inhibition of the hypoxia response pathway. Ozer et al. [118] determined that ING4 suppresses HIF-responsive genes *Nip3* and *AK3* under hypoxia without inducing the expression of HIF, suggesting that HIF activity, rather than HIF stability, is suppressed by ING4. In this regulatory process, residues 191–249 of ING4, encompassing the PHD, sufficiently interact with HPH-2C. In addition, HIF is regulated by a binding complex of ING4 and HPH in a chromatin-remodeling manner. Consistent with the evidence above, the inhibition of HIF-1α by ING4 leads to decreased IL-8 and OPN-mediated myeloma angiogenesis [48]. Furthermore, there is an extensive degree of cross-talk between NF-κB and HIF, suggesting that ING4 might inhibit the adaptation to hypoxia through NF-κB indirectly.

### Induction of cell cycle arrest, apoptosis, and autophagy

The cell cycle requires DNA replication (S phase) and the segregation of chromosomes to the daughter cells (M phase). These events are spaced by intervals of growth and reorganization (G_1_ and G_2_ phases). During cancer progression, cells are conferred with the capacity to proliferate independent of growth-inhibitory signals [119,120]. ING4 appears to be capable of conferring anti-neoplastic effects by inducing cell cycle arrest, but this varies amongst different cell lines. In RKO cells, a human colorectal cancer cell line, ING4 overexpression resulted in a decreased population of cells in the S phase and increased proportion of cells in G_1_/S and G_2_/M phases [3]. Zhang et al. [40] reported that ING4 can inhibit cell proliferation by inducing G_2_/M arrest in a dose-dependent manner in HepG2 cells by regulating the G_2_ checkpoint. This arrest is inducible through up-regulation of p21 in a p53-dependent manner. ING4-expressing recombinant adenoviral vectors in human pancreatic and lung carcinoma cell lines alter the cell cycle with a reduction in S-phase and arrest of the G_2_/M phase [99,100]. Constitutive overexpression of ING4 in human lung adenocarcinoma A549 cells significantly up-regulates the expression of p27 and down-regulates the expression of cyclin D1 and SKP2, suggesting that ING4 might inhibit cell proliferation by arresting cell cycle progression in late G_1_ via the Wnt-1/β-catenin signaling pathway [90]. The same cell cycle regulators (p27, cyclinD1, and SKP2) were also found to be involved in progression of the cell cycle in the melanoma cell line M14 [94]. In a glioma model, G_1_/S arrest was induced [85]. The cell cycle arrest differs in breast carcinoma cell lines, with the G_2_/ M phase being arrested in MDA-MB-231 cells, and a reduced S phase and arrest of the G_0_/G_1_ phase in MCF-7 cells [101,121]. Interestingly, S phase reduction and G_0_/G_1_ arrest also occur in osteosarcoma cells [51]. Qu et al. [79] detected the up-regulated expression of P21 as well as the down-regulated expression of cyclin E, demonstrating the molecular mechanism underlying ING4-induced G_1_ arrest in colorectal LoVo cells. A recent study in chronic lymphocytic leukemia cells indicates that the altered expression level of ING4 contributes to regulation of the cell cycle, thereby disrupting mitosis [76]. Nevertheless, some studies have failed to show any relevant effect of ING4 in some cell lines [5,52]. Although the role of ING4 in cell cycle control has been elucidated, it is still not entirely clear how these mechanisms contribute to ING4-mediated tumor suppression *in vivo*. As ING4 has additional functions that may directly regulate tumor suppression, the extent to which this mechanism influences tumor behavior should be investigated further.

Apoptosis is a selective physiological process of cell deletion, whereby cells undergo programmed death to eliminate damaged or harmful cells and restrict cell proliferation. The failure of apoptosis is thought to lead to the development of human malignancies. Studies have shown that a significantly higher number of RKO cells transfected with pcDNA3.1-ING4 undergo apoptosis compared with RKO cells transfected with pcDNA3.1. Meanwhile, no apoptotic induction is observed in RKO-E6 cells, a cell line with its p53 inactivated by ubiquitin-dependent cleavage that is mediated by the E6 protein of human papilloma virus, indicating that the induction of apoptosis occurs in a p53-dependent manner [3]. In addition, ING4 can also trigger apoptosis in several other cancer cell lines, such as HepG2, A549, PANC-1, SW579, and SPC-A1 [40,96,99,100,109]. Intrinsic cell death is mediated by the interaction between pro-apoptotic and pro-survival proteins of the B-cell lymphoma-2 (Bcl-2) family. The ratio of these two subsets, in part, determines the susceptibility of cells to a death signal. ING4 can up-regulate the expression of Bax and down-regulate the expression of Bcl-2, which causes the release of Cyt-*c* from the mitochondrion and the subsequent activation of caspase-3 by its cleavage, revealing that activation of the mitochondrial-induced apoptotic pathway may be involved in apoptosis of melanoma cells [94]. The high expression of p27, Bax, and Cyt-*c*, along with the low expression of SKP2, Bcl-2, caspase 3, PARP, and Cox-2, enhances apoptosis in glioma U87MG cells via activation of the mitochondrial-induced pathway [85]. This signaling pathway is shown to be involved in other tumor types as well, including lung adenocarcinoma, breast carcinoma, hepatocarcinoma, non-small-cell lung cancer, and osteosarcoma [51,101,102,121–124]. In addition to the mitochondrial-dependent intrinsic apoptosis pathway, ING4 can also trigger apoptosis through extrinsic pathways. Increased levels of Fas, cleaved caspase-8, and caspase-3, and decreased levels of FasL and procyclic acidic repetitive protein are all observed in melanoma, demonstrating that ING4 achieves functionality via the Fas/Caspase-8 pathway [125]. ING4 also initiates apoptosis in human melanoma A375 cells, which subsequently employs a classic extrinsic apoptotic pathway, the Fas/FasL-mediated signaling cascade [126]. Furthermore, oncogenic Ras regulates the ING4–thymine-DNA glycosylase (TDG)–Fas axis to trigger apoptosis in pancreatic cancer cell lines [127]. However, consistent with the abovementioned viewpoint regarding cell cycle arrest, ING4 has no apparent direct effect on cell apoptosis in other particular cell types [50]. In general, these findings indicate the important role played by ING4 in the initiation of apoptosis. Control of ING4 expression is, therefore, an important mechanism for the modulation of programmed death.

Autophagy, a lysosomal-dependent pathway, is a crucial self-catabolic process for depredating cells and for recycling cellular components. It not only serves as a survival mechanism against nutrient shortages, but also paradoxically acts as a route for cancer cell death. Gong et al. demonstrated that in glioma cells, enhanced fluorescence intensity of monodansylcadaverine (MDC) and increased expression levels of the LC3-II and Beclin-1 are related to elevated levels of autophagy [128]. Collapse of the mitochondrial membrane potential and intracellular reactive oxygen species (ROS) indicates that mitochondrial dysfunction, such as mitophagy, may be responsible for autophagic cell death. Finally, by analyzing the relationship of protein levels amongst Bax, Bcl-2, Beclin-1, and the caspase family proteins, it was confirmed that long-term treatment with ING4 can induce parallel pathways of both autophagy and apoptotic cell death in glioma cells [128]. Another study illustrates the role of endogenous ING4 as a repressor of autophagy and shows that it activates autophagy through activation of the lipid kinase activity of the Beclin 1 (BECN1)/phosphatidylinositol 3-kinase catalytic subunit type 3 (PIK3C3) complex [129].

These findings indicate that ING4 regulates many key aspects of tumorigenesis. Considering the complex mechanisms implicated in this process, we suggest that ING4 is likely to trigger multiple signaling pathways through chromatin remodeling and binding to diverse sets of proteins simultaneously. The process should be considered as one with an integrated network rather than isolated components. Multiple regulatory mechanisms observed within the same tumor model provide evidence for this statement. This may be partially attributed to structure and various splice variants of ING4. ING4 in the nucleus acts as a regulatory protein by directly interacting with chromatin as well as transcription factors. At the same time, the variant can also bind to its cytoplasmic binding partners. However, based on knowledge of other TSGs (e.g., P53) [130], this regulatory mode may be changed or disrupted under certain circumstances. More issues like this warrant further investigation.

## Potential approaches in tumor therapy

A growing understanding of approaches related to gene therapy is giving rise to novel therapeutic cancer treatments in the clinic with remarkable efficacy against tumors. For instance, adenovirus-mediated gene therapy provides an innovative therapeutic method for cancer treatment. Ad-ING4 gene transfer significantly induces tumor growth suppression and apoptosis and reduces tumor vessels and microvessel density in human osteosarcoma, lung, pancreatic, and breast carcinomas [99–102]. The same effect is achieved in human hepatocarcinoma, with Ad-ING4-mediated gene transfer in combination with chemotherapy using the drug cisplatin, with no overlapping toxicities being observed [123]. Cancer treatment is often limited due to the development of diverse multidrug resistance (MDR); however, ING4 overexpression enhances the sensitivity to cancer chemotherapies. ING4 is proven to be a modulator of docetaxel (DTX) and paclitaxel sensitivity to overcome drug resistance in human lung adenocarcinoma and colorectal cancer [91,105]. Ad-ING4 reverses gastric cancer MDR *in vitro* and *in vivo* via the down-regulation of ATP-binding cassette transporters and activation of apoptotic pathways [131]. In addition to chemotherapies, radiotherapy is also an important tool in the treatment of cancers. However, radiotherapy treatment regimens are often ineffective in clinical practice, largely due to tumor radioresistance. In studies using functional genomic screening of mutant mouse embryonic stem cells, ING4 is reported to be implicated in enhanced radiation responses [132]. In pancreatic and non-small-cell lung cancer, enhanced antitumor effects elicited by Ad-ING4, in combination with radiotherapy, have been reported to be synergistically closely connected to the activation of apoptotic pathways and inhibition of tumor angiogenesis [96,124,133].

Strategies using multigene-based combination therapy hold significant promise for developing the most effective therapeutic outcomes. While reducing drug resistance of cancer cells and toxicity to non-cancerous cells, this approach would stimulate the patient’s defensive system, leading to diminished cancer progression. For instance, IL-24, a promising candidate for cancer gene therapy, preferentially inhibits growth and induces apoptosis in a variety of cancer cells without harming normal cells [134,135]. Extensive studies have established the potent role of Ad-ING4-IL-24, at times combined with radiotherapy, in growth inhibition, invasion suppression, apoptosis induction, and inhibition of angiogenesis in cancer therapies [136–139]. Nevertheless, the combined CRAd-IL24 and CRAd-ING4 vectors demonstrate no synergistic effects exceeding the oncolytic potency of a single CRAD-IL24 vector [140]. In contrast, recombinant adenoviruses, co-expressing *ING4* along with a single gene such as *PTEN, P53*, or *OSM*, have shown a synergistic tumor-suppressive capacity in diverse cancers, including nasopharyngeal carcinoma, hepatocellular carcinoma, hypopharyngeal cancer, breast cancer, gastric cancer, and glioma, while simultaneously promoting chemosensitivity of hypopharyngeal cancer [141–148]. Furthermore, other oncolytic virus-mediated gene therapy has exhibited ubiquitous antitumor potential. A novel variant of the replication-competent oncolytic herpes simplex virus HSV1716 that expresses Ing4 (HSV1716Ing4) more efficaciously enhances the oncolytic potency of HSV1716 alone during infection of human tumor cells, both *in vitro* and *in vivo* [149]. A novel oncolytic vaccinia virus harboring *ING4* (VV-ING4) also exhibits great cytotoxic efficiency, by induction of cell cycle arrest and apoptosis in pancreatic cancer cells, amongst others; the combination of VV-ING4 and gemcitabine demonstrates synergistic effects *in vitro* and *in vivo* [150]. A newly constructed delivery system, DGL-PEG-LNP and polyethyleneimine (PEI)-grafted oxidized mesoporous carbon nanospheres (OP), can be used to efficiently deliver the therapeutic *ING4* gene (*pING4*) to tumors for gene therapy [151,152].

ING4 has also been linked to another cellular regulator, miRNAs; these are small non-coding RNAs that regulate gene expression post-transcriptionally by interfering with the translation of one or more target mRNAs. miRNAs have been reported to play key roles in diverse biological processes pertaining to cancer, including tumor growth, metastasis, angiogenesis, and drug resistance, highlighting their potential roles in therapeutic intervention against tumors [153,154]. Amongst the dysregulated miRNAs, miR-650 has been most widely discussed. miR-650 expression altered through its complementary binding to the 3′-UTR of ING4 is found to be associated with numerous cancer types, including gastric, chronic lymphocytic leukemia, lung, hepatocellular, osteosarcoma, and breast cancers [89,92,155–158]. In addition, miR-214 has been implicated in pancreatic cancer, affecting tumor growth and the response of cancer cells to chemotherapy by targetting ING4 [159,160]. Recently, three more miRNAs (miR-761, miR-330, and miR-423-5p) have been described to function as oncogenes in several cancers by suppressing ING4, suggesting their exploitation in the development of promising new therapeutic targets [84,161,162].

Thus far, all the reported *in vivo* experiments have been limited to athymic mouse models. Using ING4 gene delivery indeed showed much better therapeutic efficacy when compared with traditional chemotherapy or radiotherapy. Although promising, the development of ING4 gene therapies is still in infancy and facing numerous challenges. The first concern is the complexity of tumor tissues and cell types, which differs greatly between animal models and the human body. Another obstacle is the delivery systems. In the case of viral gene delivery, aspects such as tropism and specificity, non-toxic dose titration, off-target effects or immune responses against viral antigens require further investigation. Recently, although carbon nanospheres have been proven effective in nude mice, whether the effects will be emulated in clinical trials is still an open question. In summary, the present study is a proof-of-principle. ING4 gene therapy remains a potentially viable, yet underdeveloped, treatment for cancers, and extensive preclinical and clinical trials need to be performed.

## Impact on human disorders other than cancer

Although ING4 has been reported mostly as a TSG, it is inconceivable that ING4, a protein that regulates multiple cellular processes, is only involved in tumor suppression. Recent scientific developments have greatly expanded our knowledge of ING4 in some non-neoplastic diseases in multiple organ systems, including respiratory, cardiovascular, urinary, immune, nervous system, and skin. Interestingly, the function of ING4 shares similarities with that in tumor development, including regulation of growth, proliferation, migration, and cell death. ING4 either regulates chromatin modifications or acts as a transcription factor to regulate other proteins (e.g., NF-κB). Here, we outline some evidence that abnormalities in ING4 expression and function contribute to the etiology of other non-neoplastic diseases (Table 2).

**Table 2:** Summary of studies reporting ING4 in various non-neoplastic disorders

Organ involvement	Disorders	Origin	Methods of detection	Alteration	Possible functional mechanisms	Refs.
Respiratory system	Idiopathic pulmonary fibrosis	Patients	RT-qPCR, IHC, tissue microarrays	Down-regulation	Induce aberrant vascular remodeling, fibroblast proliferation and migration	[163]
	Cryptogenic organizing pneumonia	Patients	IHC	Down-regulation in Masson bodies		[163]
	Pulmonary sarcoidosis	Patients	RT-qPCR	Up-regulation	HIF-1a-VEGF-ING4 axis	[164]
Cardiovascular system	Ischemia/reperfusion injury	Cells	RT-qPCR	Up-regulation	Induce apoptosis	[165]
	Hypothermia	Cells	Microarray, Bioinformatics analysis	Not mentioned	Act as the transcription factor	[166]
Urinary system	K-deficient model	Rats	WB	Up-regulation in renal cortex and outer medulla	Low K intake increased ING4, suppressed ROMK channels by MAPK stimulation	[168]
Immune system	ANCA-associated vasculitis	Patients	Microarray, RT-qPCR	Down-regulation	Regulate chromatin modifications	[169]
Skin	Chronic idiopathic (spontaneous) urticaria	Patients	Microarray	Down-regulation	Cellular growth and proliferation	[170]
Nervous system	Maternal nematode infection	Mice	Next-generation sequencing, RT-qPCR	Up-regulation	Inhibit the p65 subunit of the NF-κB heterodimer to suppress innate immunity	[171]

Abbreviations: ANCA, anti-neutrophil cytoplasmic autoantibody; IHC, immunohistochemistry; MAPK, mitogen-activated protein kinase; ROMK, renal outer medullary K.

### Disorders in pulmonary and cardiovascular system

To date, pulmonary diseases have been studied the most. Tzouvelekis et al. [163] demonstrated, for the first time, the down-regulation of ING4 in a bleomycin (BLM)-induced model, and two different types of human pulmonary fibrosis, idiopathic pulmonary fibrosis (IPF) and cryptogenic organizing pneumonia (COP). They claimed that the reduced expression of ING4 may facilitate aberrant vascular remodeling, and fibroblast proliferation and migration, which leads to progressive disease, culminating in a fatal outcome. In another report, Piotrowski et al. [164] observed abundant ING4 expression from samples of bronchoalveolar lavage fluid (BALF) and peripheral blood of pulmonary sarcoidosis patients. In their view, ING4 probably affects angiogenesis and lung epithelial remodeling through the HIF-1a-VEGF-ING4 axis. Cardiac tissue, an important part of the cardiovascular system, has minimal regenerative capacity in response to injury. A trial confirmed that miR-199a-3p and miR-214 protect cardiomyocytes against simulated ischemic injury, in part via repression of ING4, thus contributing to cardiomyocyte protection induced by carvedilol [165]. In addition, ING4 serves as a transcription factor that may play an important role in regulating myocardial function in response to hypothermia [166].

### Disorders in other systems

One of the key functions of the kidney is maintaining plasma K homeostasis [167]. In renal K secretion regulation, low K intake increases ING4 expression in a superoxide-dependent manner, subsequently stimulating mitogen-activated protein kinase (MAPK) to depress renal outer medullary potassium (ROMK) channel activity [168]. Furthermore, ING4 was found to play an active role in the multi-system autoimmune disease. Based on the reduced ING4 expression in leukocytes from anti-neutrophil cytoplasmic autoantibody (ANCA)-associated vasculitis (AAV) patients and the acetylation of histone H4K16 modified by complexes containing ING4, Yang et al. [169] suggested that the epigenome may be involved in AAV pathogenesis. This functional role is similar to that observed in tumors on the level of epigenetic modification. However, the actual mechanism of ING4 in establishing and maintaining chromatin modifications remains uncertain. ING4 is also found to be differentially expressed in patients with chronic idiopathic urticaria and in the fetal brains of mice in response to maternal nematode infection [170,171]. During fetal liver erythropoiesis, bromodomain-containing protein 1 (BRD1) forms a complex with HBO1 and ING4 and plays a crucial role in the transcriptional activation of key developmental regulator genes [172]. The above studies highlight the emerging role of ING4 beyond the field of cancer.

Although these initial results are promising, few studies are currently at the stage of bioinformatics analysis. Several underlying mechanisms remain unclear and need further confirmation; nevertheless, these studies provide a basis for exploring ING4 as a potential therapeutic target in non-neoplastic diseases.

## Conclusions and future perspectives

ING4, a protein involved in tumorigenesis, has emerged as an attractive topic for further study aimed to better understand cancer biology. The protein structure and specific genetic splice variants of ING4 give the protein its unique features and variability. Initial researches regarding ING4 focussed on exploring its ability to remodel chromosomes. Researchers have gradually become aware of the associations of ING4 with P53, NF-κB, HIF, and MYC in regulating various cell signaling pathways. In some types of human cancers, ING4 exerts its function through regulation of cell cycle arrest, apoptosis, autophagy; tumor angiogenesis, metastasis, invasion; intercellular contact inhibition; and suppression of adaptation to hypoxia. In addition, the role of ING4 in non-neoplastic diseases is gradually attracting attention. The most recent developments of ING4 are related to its application in Ad-ING4 TSG therapy, sometimes in combination with radiation or chemotherapy to enhance its therapeutic effect.

There are still many interesting questions and knowledge gaps that need to be addressed in future studies. Does the lack of ING4 expression also exist in other caners? What are the precise mechanisms that ING4 utilizes to regulate these cellular processes and signaling molecules? Is there any other non-neoplastic disease that is closely related to ING4 deficiency? Will restoration of ING4 expression prove to be an efficient means for treating cancer patients? As most previous studies have focussed on the downstream factors of ING4, the exact molecular mechanism that initiates ING4 down-regulation remains unclear. A better understanding of the upstream regulator of ING4 is important for providing better insights into restoring the ING4 tumor-suppressor function and will lead to the development of specific strategies for treatment. Several mechanisms, such as epigenetic modifications and miRNAs, are proven to be involved in the regulation of ING4. We speculate that there are other factors that affect ING4 expression, such as the environment. Further studies are needed to investigate the potential of ING4 as a cancer biomarker as well as its therapeutic strategies by using clinical samples.

## Abbreviations

AAV: ANCA-associated vasculitis
Ad-ING4: adenovirus-mediated *ING4*
AUF1: AU-rich RNA-binding factor 1
Bcl-2: B-cell lymphoma-2
BECN1: Beclin 1
EMT: epithelial–mesenchymal transition
HAT: histone acetyltransferase
HBO1: histone acetyltransferase binding to ORC1
HIF-1α: hypoxia inducible factor-1 α
HPH-2: HIF prolyl hydroxylase-2
H3K4me3: histone H3 trimethylated at lysine 4
IL: interleukin
ING4: inhibitor of growth 4
IκB: inhibitor of κB
LZL: leucine zipper-like
MDR: multidrug resistance
MMP: matrix metalloproteinase
NCR: novel conserved region
NF-κB: nuclear factor κB
OPN: osteopontin
PHD: plant homeodomain
PIK3C3: phosphatidylinositol 3-kinase catalytic subunit type 3
ROS: reactive oxygen species
TDG: thymine-DNA glycosylase
TSG: tumor suppressor gene
TSS: transcription start site
VEGF: vascular endothelial growth factor
VV-ING4: vaccinia virus harboring *ING4*
